# Non-specific Low Back Pain Among Nurses in Qassim, Saudi Arabia

**DOI:** 10.7759/cureus.19594

**Published:** 2021-11-15

**Authors:** Abeer Abuzeid Atta Elmannan, Hajar A AlHindi, Reema I AlBaltan, Mariah S AlSaif, Nouf S Almazyad, Ruba K Alzurayer, Shouq Al-Rumayh

**Affiliations:** 1 Family and Community Medicine, College of Medicine, Qassim University, Buraidah, SAU; 2 Medicine, College of Medicine, Qassim University, Alrass, SAU; 3 Family and Community Medicine, College of Medicine, Qassim University, Alrass, SAU; 4 Medicine, College of Medicine, Qassim University, Buraidah, SAU; 5 Anesthesia, College of Medicine, Qassim University, Alrass, SAU; 6 Medical Intern, College of Medicine, Qassim University, Buraidah, SAU

**Keywords:** saudi arabia, qassim, risk factors, prevalence, low back pain

## Abstract

Introduction

Non-specific low back pain (LBP) is a complex and multifactorial health problem. Evidence has shown that LBP is an important occupational hazard and nurses are particularly at high risk. While several studies have addressed the prevalence of LBP worldwide, the prevalence of LBP in Saudi Arabia remains unclear. In this study, we aimed to estimate the prevalence and associated factors of LBP among nurses in the Qassim region, Saudi Arabia.

Methods

This was a multicenter cross-sectional study carried out in four major public hospitals in the Qassim region. A total of 323 nurses were recruited through a two-stage sampling method. A previously validated questionnaire was used to gather data. The main outcome measures were; LBP prevalence during working life, demographic factors, lifestyle factors, work-related factors, and psychological factors. Multivariable logistic regression analysis was used to determine factors independently associated with LBP.

Results

The study showed that LBP prevalence was 65.6% (n=212). Over one-third of the study, participants sought treatment for LBP (n=82, 38.7%). Age and the type of ward were found significantly associated with LBP [adjusted odds ratios (aOR): 0.39; 95% confidence interval (CI): 0.19, 0.77; p value=0.007] & (aOR: 0.36; 95% CI: 0.15, 0.86; p-value =0.02), respectively. However, gender, working hours, number of patients, stress, and smoking were not identified as LBP risk factors in this study.

Conclusion

The findings of this study suggest that LBP is a highly prevalent occupational health problem among nurses in Qassim. Young nurses 20-30 years are more likely to suffer from LBP, while nurses working in the general surgery wards have a lower risk for LBP in this study. On-the-job training is essential particularly for new and young nurses on proper body mechanics when mobilizing patients or lifting heavy equipment. In addition, there is a need for evidence-based interventions to improve the workplace environment for nurses in hospitals in order to lower LBP prevalence.

## Introduction

Low back pain (LBP) is a common complaint that needs medical attention. Indeed, LBP is the commonest musculoskeletal disorder among adults with a prevalence reaching 84% [1]. It has a complex etiology and may originate from different spinal structures including muscles and fascia, joints, ligaments, discs, or nerve roots [2]. However, in many cases, no definitive cause can be determined. Non-specific LBP is a term used when the underlying cause cannot be specifically identified [1]. The diagnosis of non-specific LBP is generally made upon the exclusion of other known causes for the LBP such as infections, trauma, or neoplasms [3]. It commonly refers to pain localized in the posterior region of the body extending from below the costal margin down to the gluteal folds with or without referred pain into one or both legs, lasting for at least one day [4]. Throughout this paper, we will use the abbreviation LBP to refer to non-specific low back pain.

LBP may result in major economic losses. Health care expenditure on LBP in terms of costs of care and treatment is significant [5]. Prior evidence has shown that recurrent back pain was strongly associated with increased odds of leaving paid employment for health-related reasons [6]. LBP may also lead to many physical and psychological complications. Owing to the nature of their job nurses are considered among the groups at high risk for LBP [7-9]. Several studies identified work-related factors such as lifting and mobilizing patients, inappropriate work design, sustained postures, decreased social support, low job satisfaction, and inadequate staffing as the main risk factors for LBP among nurses [10-12]. It has also been found that LBP adversely influences the productivity of nurses and thus undermines the overall quality of healthcare services provided to beneficiaries [13]. Moreover, LBP has a great impact on the health care system, particularly in terms of work absenteeism, and escalating healthcare costs [14].

Psychosocial factors were also found to be linked with the risk for LBP [15]. The increased demand of the job in terms of prolonged working hours, night shifts, spending a long time away from their families may negatively impact the psychological status of nurses. In addition, low job satisfaction, increased workload, and poor work relationships may result in stress and anxiety [16,17].

A little is known about the burden of LBP among nurses in Saudi Arabia. A previous study on the prevalence of LBP among physical therapists revealed a high prevalence of 89.65%. The study also found that gender and duration of patient contact were associated with LBP [18]. Another study which was conducted among health care workers in the southwestern region of Saudi Arabia estimated LBP as 73.9% [19]. Furthermore, a community-based survey that was carried out in Qassim has only focused on the prevalence of back pain among the general population. Over 5000 adults participated in the survey which reported a relatively low prevalence of 18.85%. The survey also showed that age, depression, and certain occupations were associated with back pain [20]. A few researchers have addressed the problem of LBP in Saudi Arabia. This raises questions about how big this problem is among healthcare professionals and particularly among nurses. The sacristy of evidence for LBP in Saudi Arabia was the main driving force to carry out this investigation. In this study, we aimed to determine the prevalence of low back pain among nurses working in the public health sector in Qassim in order to gain insight into the magnitude of this problem and its predicting factors.

This article was posted to the Research square preprint server on August 18, 2020.

## Materials and methods

Study setting

This was a multicenter cross-sectional study carried out in the Qassim region in Saudi Arabia. In this study, four major public hospitals representing the four major cities in Qassim were included, namely; King Fahd Specialist Hospital (126 nurses), Ar Rass General Hospital (96 nurses), King Saud Hospital (49 nurses), and Al Bukairiyah General Hospital, (52 nurses).

Study population

A total of 323 nurses were recruited in this study. A two-stage sampling technique was employed. In the first stage, hospitals were selected (primary sampling units). Eligible hospitals were public facilities that provide inpatient care and admit all types of cases. Four hospitals serving large catchment areas in Qassim were included. Secondly, a sample of professional nurses was recruited from each hospital (secondary sampling units). Eligible nurses were registered nurses from both sexes who were on full-time jobs and were on duty during the data collection period. Those who were on official leave were excluded. We calculated the sample size using the single population proportion equation (N=Z2 p (1-p) / d2), where;

N: Target sample size

Z: The standard normal variant at 5% type 1 error (p value < 0.05), which is 1.96.

P: Expected prevalence of low back pain among nurses obtained from previously published studies.

D: Absolute error or precision, decided as 5%.

An assumed prevalence of LBP of 70% was used based on previous evidence [19,21,22], a precision level of 5%, and 95% confidence level. That yielded the target sample size of 323 nurses.

The questionnaire

The prevalence of low back pain and other related data were measured through the administration of an anonymous, self-administered questionnaire. The questionnaire was adapted from a previously published study by Branny and Newell, and we used it unchanged [23]. It is composed of four parts; job-related history, LBP characteristics, LBP treatment options, and socio-demographic factors. LBP was defined with an aid of a diagram to depict the area of pain localization. The definition and illustration used in this questionnaire were the same used in other British surveys on back pain [21]. Questionnaires were hand distributed, and informed consent was obtained from nurses who agreed to take part in the study. The study protocol was reviewed and approved by the regional institutional review board in Qassim.

Data analysis

The statistical analysis was performed in two steps. First, descriptive statistics analysis was performed. Background data related to sociodemographic risk factors, work history, and prevalence of LBP were presented as simple frequencies (and percentages). Secondly, inferential statistics in the form of logistic regression analyses were performed. Initially univariate logistic regression was used to calculate crude odds ratios (unadjusted ORs) and determine potential risk factors for LBP separately. Next, multivariate logistic regression analysis was performed to obtain adjusted odds ratios (aORs) and 95% confidence intervals (95% CI) to identify variables significantly associated with LBP considering the effect of potential confounders.

Ethics approval and consent to participate

The study has been approved by the Regional Research Ethics Committee under the General Directorate of Health Affairs/ Ministry of Health in Qassim Province. It has been registered at the National Committee of Bio.& medical ethics in Saudi Arabia (Reference Number: H-04-Q-001). Participation was voluntary, and written consent was obtained from participants prior to completing the questionnaire.

## Results

In this study, a total of 323 questionnaires were distributed. All participants completed the questionnaire resulting in a response rate of 100%. For ease of administration, all questionnaire items were closed-ended. In Table 1 the socio-demographic profile of participants was described. Females comprised the vast majority among participants (n=302, 93.5%). Most participants (n=195, 60.4%) belong to the age group 20-30 years. Smoking habit was observed among only 5% (n=16) of the participants. Regular physical activity was reported by 31.0% (n=100) of participants. Feeling low in mood or under stressed were reported by 77.1% and 82.9% of participants, respectively.

**Table 1:** Demographic & life style characteristics of nurses, Qassim, Saudi Arabia (n=323)

Characteristic	N (%)
Gender	
Male	21(6.5%)
Female	302 (93.5%)
Age group	
20- 30 years	195 (60.4%)
31-40 years	99 (30.7%)
Over 40 years	29 (9.0%)
Smoking	
No	307 (95%)
Yes	16 (5.0%)
Regular Physical activity	
No	223 (69.0%)
Yes	100 (31.0%)
Feeling low in mood	
Never	74 (22.9%)
Occasionally	215 (66.6%)
Frequently	34 (10.5%)
Feeling under stress	
Never	55 (17.0%)
Occasionally	202 (62.5%)
Frequently	66 (20.4%)

Table 2 shows the work history of the participants. The majority of participants in this study were staff nurses (n=265, 82.0%). Charge nurses and healthcare assistants comprised 17.1% and 0.9% respectively. A total of 183 (56%) of the study participants spent one to five years working in their current post. More than two-thirds of the enrolled nurses (n= 254, 78.6%) reported working on day and night shifts alike. The majority of nurses (n=262, 81.1%) work over 40 hours per week. Moreover, 140 (43.3%) of participants had to work additional hours exceeding 10 hours on average per month. On a regular shift, more than half of participants (n= 187, 57.9%) reported an average of one to five patients requiring assistance mobilizing, while 34.4% reported a higher number exceeding five patients per shift. Nurses in this study worked in a wide variety of wards, however, at the time of the survey a considerable number of them worked in three main wards; general medical, intensive care, and general surgical ward comprising 19.8%21.7% and 18.9% respectively.

**Table 2:** Work-related characteristics of nurses, Qassim, Saudi Arabia (n=323)

Characteristic	N (%)
Current post	
Healthcare assistant	3 (0.9%)
Staff nurse	265 (82.0%)
Charge nurse /sister	55 (17.1%)
Work duration in the current post	
less than one year	41 (12.7%)
1-5 years	183 (56.7%)
More than 5 years	99 (30.7%)
Type of shifts	
Days only	67 (20.7%)
Nights only	2 (0.6%)
Days and nights	254 (78.6%)
Work hours per week	
20- 40	61 (18.9%)
Over 40 hours	262 (81.1%)
Additional work hours per month	
None	41 (12.7%)
1-10	142 (44.0%)
More than 10	140 (43.3%)
Average number of patients require assistance mobilizing per shift	
None	25 (7.7%)
1-5	187 (57.9%)
Over 5 patients	111 (34.4%)
Type of ward	
General medical	64 (19.8%)
ICU	70 (21.7%)
General surgical	61 (18.9%)
Others	128 (39.6%)

The prevalence of LBP and its related characteristics are shown in Table 3. This study showed a 65.6% prevalence of LBP among nurses in Qassim. Over one-third of them sought treatment for LBP (n=82, 38.7%). Similarly, at least one-third reported difficulty performing daily activities such as getting out of bed (47.2%), sleeping through the night (48.1%,), and putting on socks (37.7%). However, only a few reported sickness absences due to LBP (n=65, 31.7%).

**Table 3:** Prevalence of low back pain among nurses, Qassim, Saudi Arabia (n=323)

Characteristic	N (%)
Low back pain	212 (65.6%)
Treatment sought for low back pain	82 (38.7%)
Low back pain Spread down the leg to below the knee	131 (61.8%)
Low back pain causing difficulty in daily activities below	
Getting out of bed	100 (47.2%)
Sleep through the night	102 (48.1%)
Standing up for 20-30minutes.	121 (57.1%)
Walking 300-400 meter	107 (50.5%)
Climb one flight of stairs	107 (50.5%)
Put on socks	80 (37.7%)
Sickness Absence due to low back pain	
None	147 (68.3%)
1-6 days	42 (19.8%)
1-4 weeks	15 (7.0%)
more than 4 weeks	8 (3.8%)

Table 4 describes the univariate and multivariate logistic regression analysis of factors significantly associated with LBP. The univariate analysis showed that working on a general surgical ward (OR: 0.39; 95% CI: 0.21, 0.73; p-value = 0.004) ,and age over 40 years (OR: 0.35; 95% CI: 0.16, 0.78; p-value = 0.01) were significant factors. In the multivariate logistic regression analysis, the same variables were also found independently associated with LBP expressed as; working on a general surgical ward (aOR: 2.9; 95% CI: 0.19, 0.77; p-value = 0.007), and age over 40 years (aOR: 0.36; 95% CI: 0.15, 0.86; p-value = 0.02). However, smoking, physical activity, gender, work duration, length of working hours per week, number of patients requiring assistance mobilizing on a shift, depression, and stress were not independent risk factors for LBP (p-value ≥ 0.05).

**Table 4:** Univariate & Multivariate logistic regression analysis of factors associated with low back pain among nurses, Qassim, Saudi Arabia (n= 323) * Significant; UOR: unadjusted odds ratio; AOR: adjusted odds ratio

Characteristic	Low Back Pain		UOR (95% CI)	p.value		AOR (95% CI)	p.value
	Yes(%)	No (%)					
Working hours per week							
20-40	46 75.4%	15 24.6%	1			1	
More than 40	166 63.4%	96 36.6%	0.564 (0.299 -1.064)	0.077		0.628 (0.314 -1.256)	0.189
Work duration in current post							
Less than one year	26 63.4%	15 36.6%	Reference			Reference	
1-5 years	119 65.0%	64 35.0%	1.073 (0.530 -2.170)	0.845		0.995 (0.467 -2.120)	0.990
More than 5 years	67 65.6%	32 34.4%	1.208 (0.564 -2.589)	0.627		1.425 (0.608 -3.337)	0.415
Additional work hours per week							
None	31 75.6%	10 24.4%	Reference			Reference	
1-10	92 64.8%	50 35.2%	0.594 (0.269 -1.310)	0.197		0.581 (0.241-1.403)	0.228
More than 10	89 63.6%	51 36.4%	0.563 (0.255 -1.242)	0.155		0.531 (0.217 -1.301)	0.166
Type of ward							
General medical	40 62.5%	24 37.5%	0.627 (0.331 -1.187)	0.152		0.669 (0.335 -1.334)	0.254
ICU	48 68.6%	22 31.4%	0.821 (0.434 -1.552)	0.544		0.835 (0.426 -1.637)	0.599
General surgical	31 50.8%	30 49.2%	0.389 (0.206 -0.734)	0.004*		0.387 (0.194 -.772)	0.007*
others	93 72.7%	35 27.3%	Reference			Reference	
Patients require mobilizing							
None	16 64.0%	9 36.0%	Reference			Reference	
1-5	119 63.6%	68 36.4%	0.984 (0.413 -2.348)	0.972		1.207 (0.437 -3.331)	0.716
More than 5	77 69.4%	34 30.6%	1.274 (0.512 -3.168)	0.602		1.513 (0.521 -4.392)	0.446
Gender							
Male	14 66.7%	7 33.3%	1			1	
Female	198 65.6%	104 34.4%	0.952 (0.373 -2.432)	0.918		0.660 (0.214 -2.038)	0.470
Age group							
20- 30 years	136 69.7%	59 30.3%	Reference			Reference	
31-40 years	63 63.6%	36 36.4%	0.759 (0.455 -1.265)	0.291		0.645 (0.356 -1.168)	0.148
Over 40 years	13 44.8%	16 55.2%	0.352 (0.159 -0.779)	0.010*		0.356 (0.148 -.860)	0.022*
Smoking							
No	203 66.1%	104 33.9%	1			1	
Yes	9 56.3%	7 43.8%	0.659(0.239 -1.819)	0.421		0.638 (0.195 -2.082)	0.456
Regular Physical activity							
No	146 65.5%	77 34.5%	1		1		
Yes	66 66.0%	34 34.0%	1.024 (0.623 -1.683)	0.926	1.078 (0.621-1.874)		0.789
Feeling low in mood							
Never	47 63.5%	27 36.5%	Reference		Reference		
Occasionally	140 65.1%	75 34.9%	1.072 (0.619 -1.859)	0.803	1.050 (0.489 -2.252)		0.901
Frequently	25 73.5%	9 26.5%	1.596 (0.651-3.913)	0.307	1.388 (0.433 -4.453)		0.581
Feeling under stress							
Never	33 60.0%	22 40.0%	Reference		Reference		
Occasionally	132 65.3%	70 34.7%	1.257 (0.681-2.319)	0.464	1.491 (0.631 -3.523)		0.362
Frequently	47 71.2%	19 28.8%	1.649 (0.773 -3.520)	0.196	1.742 (0.609 -4.982)		0.301

## Discussion

This study aimed to objectively measure the prevalence of LBP and its risk factors among nurses in Qassim. This research is potentially the first comprehensive analysis of LBP involving four major public health facilities in Qassim. We aimed to measure the prevalence of LBP of nurses during their working life. The study found the prevalence of LBP was 65.5%. The literature shows a wide variation in LBP prevalence. However, the numbers are invariably high as reported in previous international studies conducted among nurses in Switzerland [24], Nigeria [25], Slovenia [26], Jordan [27], and South Africa [28]. Nationally, LBP prevalence was reported as 74.2%, and 48.4% among operating room staff in Makkah & Taif respectively [29,30]. In addition, a study showed a prevalence of 53.2 % among nurses in Sudayr region [31]. Those findings reflect the burden of LBP among an important healthcare workforce. However, no significant gender-related differences in prevalence were reported. This finding is inconsistent with previous studies that showed a higher prevalence of LBP among females compared to males [25,32]. Nurses are at the heart of healthcare systems worldwide and have an indispensable role in the delivery of healthcare. Our results also showed that 61.8% of nurses who reported LBP had suffered from pain spreading to below their knees and over 38% of them had sought treatment for LBP. In addition, over 31% of nurses in the study reported absence from work due to LBP. This finding is in agreement with a previous Saudi study indicating that almost 44% of nurses reported considering changing their job due to LBP [33].

An in-depth analysis of the potential risk factors for LBP shows that occupation-related back pain is a complex phenomenon and its underlying causes are multifactorial. In the literature, A web of causation of LBP was described which includes; demographical factors, lifestyle factors, occupational factors, and psychological factors [34].

Regarding the demographic characteristics and based on the logistic regression analyses, our study found that age had significantly different odds for the study participants with LBP when compared with those without LBP. Nurses aged over 40 years were less likely to develop LBP when compared to nurses of younger age. Specifically, they had 64% less likely odds of developing LBP. Our result is in contrast with other studies which show LBP is associated with older age [25,35-36]. On other hand, however, this result is supported by past evidence which indicates that younger nurses between 20-30 years had the highest prevalence of LBP [37]. This might be due to the fact that younger nurses are more likely to be involved in heavy workloads as healthcare assistants or staff nurses. This type of workload involves assistance mobilizing of patients or instruments and hence requires more musculoskeletal effort. While older, probably senior nurses are expected to be responsible for organizational or supervisory jobs with less musculoskeletal strains. This finding could also be attributed to training. Older nursing staff would have received extensive training on health and occupational safety and had developed awareness and skills on safe posture techniques over time and so less likely to adopt faulty postures. Additionally, younger nurses with limited work experience and high job demands may suffer more from psychological stress compared to older nurses who might have already developed effective strategies to cope with work and personal stress. This explanation is supported by a previous study that indicates that younger nurses have higher job-related stress [37]. Another plausible explanation for the decreased odds for LBP among older nurses is the healthy worker effect. LBP sufferers tend to change their employment or quit their jobs, whereas healthy nurses are more likely to stay in their jobs [38].

This study also investigated the effect of lifestyle factors on LBP. Evidence from the literature suggests a strong association between smoking and LBP confirming that smokers are more prone to LBP [39]. It has been found that nicotine significantly decreases the amount of oxygen reaching the muscles resulting in an increased likelihood for muscular injury and degenerative changes [40]. Additionally, it is plausible that smoking induces coughing reflexes which may further increase the risk among smokers for LBP. However, no significant effect of smoking could be detected in this study. A previous study showed similar results [41]. Historically, females in the Arab world are less likely to report their smoking habits for cultural reasons. Over 90% of the study participants were females and therefore under-reporting could be the reason why smoking couldn`t be detected as a significant predicator for LBP in this study. Similarly, no significant association was found between physical activity and LBP in this study. Interestingly, past evidence detected that physical exercise such as pilates intervention reduces LBP [42]. Some authors, however, described the relationship between physical activity and LBP as U-shaped, where moderate increased risk was exclusively found for those engaged in strenuous activities and those living a sedentary lifestyle [43].

Besides their professional duties, nurses are expected to assist in other ancillary services such as mobilizing patients or equipment. Five occupation-related risk factors were tested in this study, those were; working hours per week, work duration, additional work hours per week, type of ward, and the number of patients requiring mobilization. Of those, only the factor “type of ward” remained statistically significant after adjusting for potential cofounders. Prior evidence has shown that LBP prevalence among nurses working in intensive care units was particularly high [44]. Another study showed that nurses working in the Obstetrics and Gynecology had a high prevalence of LBP exceeding 26% [25]. A different study revealed that the prevalence of LBP was higher among nurses working in surgical wards [45]. Surprisingly, our study found that nurses working in the general surgery ward have 61% fewer odds for LBP compared to other departments. Surgical nurses are expected to be more exposed to back pain and injuries due to the nature of the work compared to nursing duties in other wards. Examples of risk include working with dependent patients, standing in one position during lengthy surgical operations, holding patient extremities, moving anesthetized patients, lifting equipment, carrying heavy trays, etc. However, nurses working in surgical departments might have already developed better awareness of this particular risk and hence have become well prepared for their jobs. They might have better skills related to body mechanics and ergonomics compared to nurses working in other wards resulting in lower risk among them. Alternatively, the significant association between working in a surgical ward and lower risk for LBP might indirectly be related to the perceived amount of workload in terms of less number of patients, and a shorter hospital stay of patients in surgical wards. In fact, it is not clear from our data the length of work experience in wards for nurses at the time of the study and its relation to the onset of LBP. Nurses typically rotate between wards during their working life and it is difficult to determine the temporal association between the type of ward and development of LBP in this study. Psychological factors were documented by other studies to have a significant association with LBP [16,33,34], however, stress and low mood were not identified as risk factors of LBP in this study.

Limitations

Our findings have provided an important scientific contribution. It clearly demonstrates that LBP is a highly prevalent health problem among nurses in Qassim. However, our work has three limitations. First, although we were able to study several variables, the temporal association could not be established between LBP and significant variables due to the cross-sectional design. Second, response bias could not be excluded since data were collected through self-reporting. The effect of this bias was minimized through the use of closed-ended, and concise answer choices in the study questionnaire. Also, the questions were direct and clearly phrased. Third, due to the small number of male participants, it was difficult to determine if there was a true gender-specific difference in LBP prevalence in this study. In general, despite limitations, we believe that our work adds substantially to a growing body of literature on the prevalence of LBP among nurses in Saudi Arabia.

## Conclusions

The evidence from this study suggests that LBP is a common occupational health problem among nurses. The high LBP prevalence reported in this study is comparable to other countries. However, targeted interventions are needed to reduce this prevalence. It has been observed that younger nurses who constitute the largest group of the ward team in any hospital are more prone to have LBP. Nurses work in direct contact with patients, and their health not only influences their job satisfaction but also patient safety and quality of healthcare. Interestingly, the results of this study showed that nurses working in general surgery wards where one would expect a heavy workload have a lower risk for LBP. In our view, these results have important managerial implications. Improving the workplace environment for nurses is essential to reduce the risk for LBP. Ergonomics research is required to identify evidence-based interventions that would help promote the health and safety of nurses, as well as on-the-job training to raise nurses’ awareness and improve their skills on how to use body mechanics and avoid risky postures.
